# 3-Chloro-2-methyl­anilinium chloride monohydrate

**DOI:** 10.1107/S1600536814009921

**Published:** 2014-05-10

**Authors:** Arbi Maroua, Khederi Lamia, Hamdi Ahmed, Rzaigui Mohamed

**Affiliations:** a Laboratory of Materials Chemistry, Faculty of Sciences of Bizerte, 7021 Zarzouna, Bizerte, Tunisia

## Abstract

In the title hydrated salt, C_7_H_9_ClN^+^·Cl^−^·H_2_O, the organic cations, anions and water mol­ecules are connected by N—H⋯Cl, N—H⋯O and O—H⋯Cl hydrogen bonds. These inter­actions lead to the formation of layers parallel to the *ac* plane.

## Related literature   

For hydrogen bonds, see: Steiner (2002 ▶); Jayaraman *et al.* (2002 ▶). For the crystal structure of a related protonated amine, see: Hamdi *et al.* (2014 ▶). For related structures containing the 3-chloro-2-methyl­anilinium cation, see: Khemiri *et al.* (2008 ▶); Bel Haj Salah *et al.* (2014 ▶). For geometrical features, see: Oueslati *et al.* (2005 ▶).
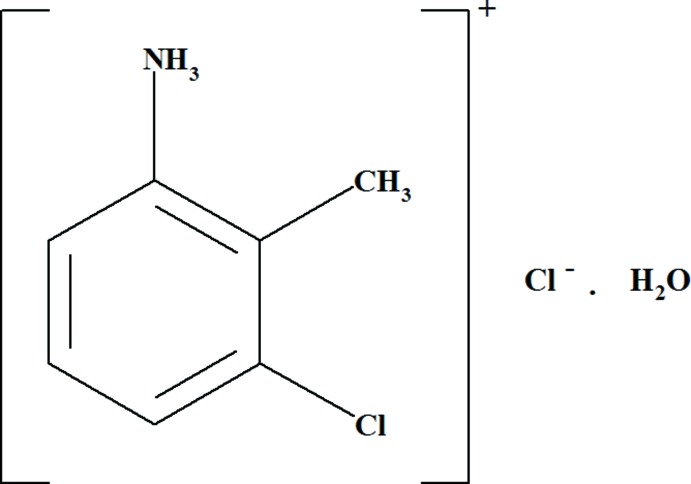


## Experimental   

### 

#### Crystal data  

C_7_H_9_ClN^+^·Cl^−^·H_2_O
*M* *_r_* = 196.07
Orthorhombic, 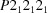

*a* = 7.434 (4) Å
*b* = 7.475 (3) Å
*c* = 16.785 (2) Å
*V* = 932.7 (6) Å^3^
*Z* = 4
Ag *K*α radiation
λ = 0.56087 Å
μ = 0.33 mm^−1^
*T* = 293 K
0.50 × 0.25 × 0.15 mm

#### Data collection  

Enraf–Nonius CAD-4 diffractometer
5019 measured reflections
3947 independent reflections
1804 reflections with *I* > 2σ(*I*)
*R*_int_ = 0.035
2 standard reflections every 120 min intensity decay: 6%

#### Refinement  

*R*[*F* ^2^ > 2σ(*F* ^2^)] = 0.055
*wR*(*F* ^2^) = 0.163
*S* = 0.92
3947 reflections
100 parameters
3 restraints
H-atom parameters constrained
Δρ_max_ = 0.35 e Å^−3^
Δρ_min_ = −0.42 e Å^−3^
Absolute structure: Flack (1983 ▶), unique data only
Absolute structure parameter: −0.04 (19)

### 

Data collection: *CAD-4 EXPRESS* (Enraf–Nonius, 1994 ▶); cell refinement: *CAD-4 EXPRESS*; data reduction: *XCAD4* (Harms & Wocadlo, 1995 ▶); program(s) used to solve structure: *SHELXS97* (Sheldrick, 2008 ▶); program(s) used to refine structure: *SHELXL97* (Sheldrick, 2008 ▶); molecular graphics: *ORTEP-3 for Windows* (Farrugia, 2012 ▶); software used to prepare material for publication: *WinGX* (Farrugia, 2012 ▶).

## Supplementary Material

Crystal structure: contains datablock(s) I. DOI: 10.1107/S1600536814009921/fj2671sup1.cif

Structure factors: contains datablock(s) I. DOI: 10.1107/S1600536814009921/fj2671Isup2.hkl

Supporting information file. DOI: 10.1107/S1600536814009921/fj2671Isup3.cml

CCDC reference: 1000637

Additional supporting information:  crystallographic information; 3D view; checkCIF report

## Figures and Tables

**Table 1:** Hydrogen-bond geometry (Å, °)

*D*—H⋯*A*	*D*—H	H⋯*A*	*D*⋯*A*	*D*—H⋯*A*
O1*W*—H1*W*1⋯Cl2^i^	0.86	2.28	3.129 (3)	169
O1*W*—H2*W*1⋯Cl2	0.86	2.36	3.139 (3)	151
N1—H1*A*⋯Cl2^ii^	0.89	2.70	3.178 (3)	115
N1—H1*A*⋯O1*W* ^iii^	0.89	2.30	2.667 (4)	105
N1—H1*B*⋯Cl2^iv^	0.89	2.68	3.187 (3)	117
N1—H1*B*⋯O1*W* ^iii^	0.89	2.28	2.667 (4)	106
N1—H1*C*⋯Cl2^ii^	0.89	2.83	3.178 (3)	105

Symmetry codes: (i) 
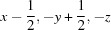
; (ii) 
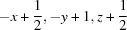
; (iii) 
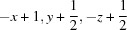
; (iv) 
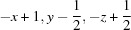
.

**Fig. 1.**
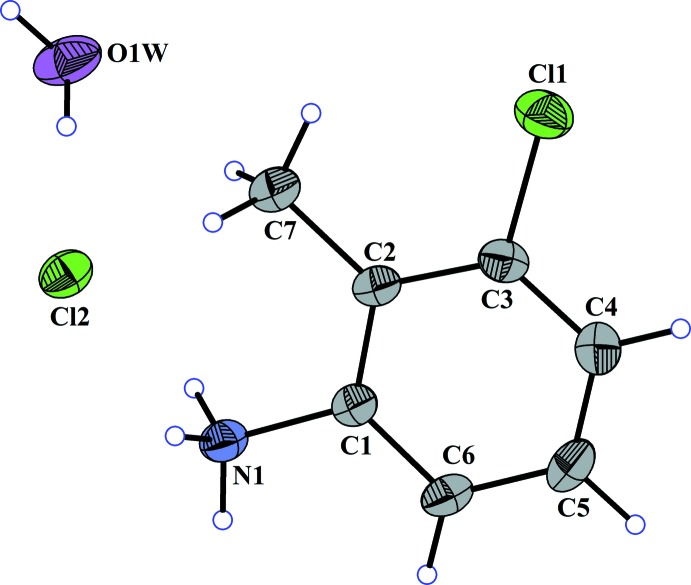
ORTEP drawing of the asymmetric unit of title compound with displacement ellipsoids drawn at 40% probability level. [Symmetry code: (i) x, y, z]

**Fig. 2.**
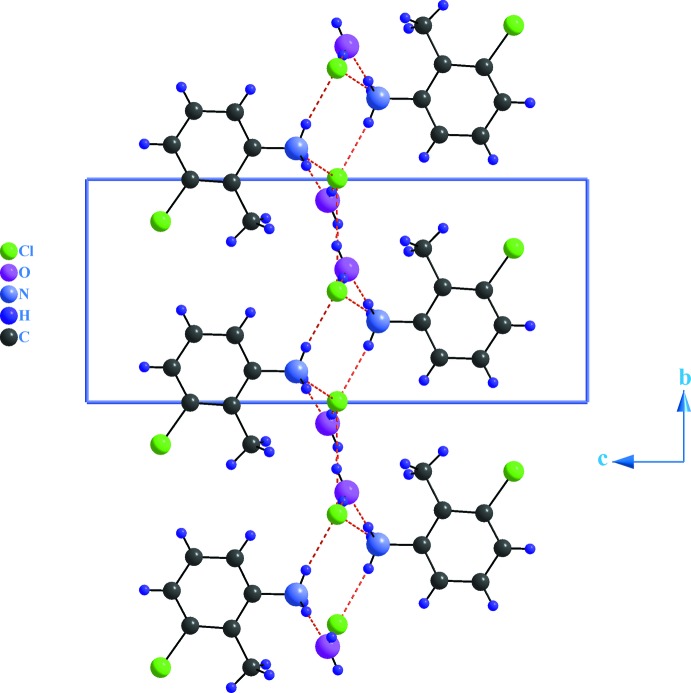
A view of the atomic arrangement of the title compound along the a axis with H bonds shown as dashed lines.

**Fig. 3.**
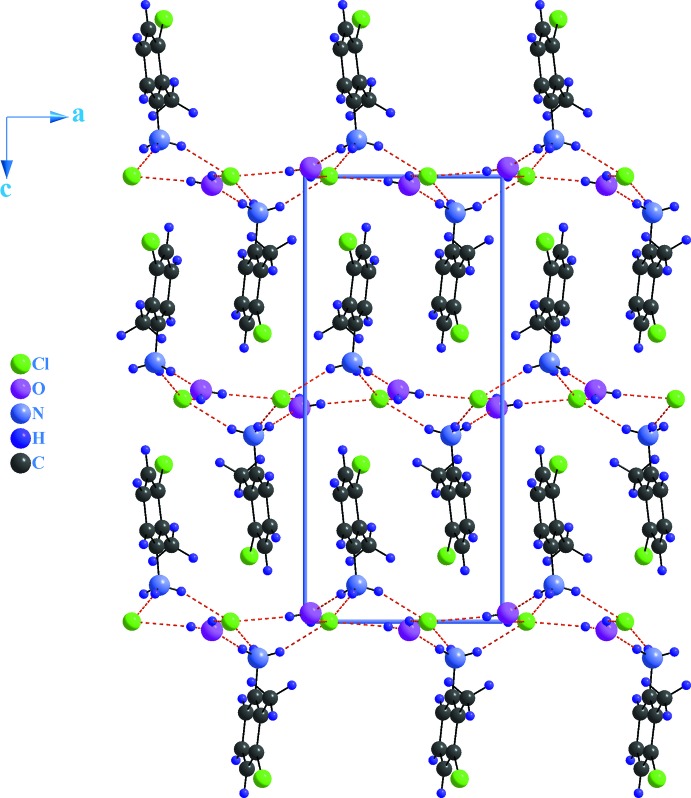
A diagram of the crystal packing in the title compound, viewed down the b axis.

## supplementary crystallographic information

### 1. Comment

Hydrogen bonding is of interest because of their prevalent occurrence in biological systems (Steiner 2002; Jayaraman *et al.*, 2002). Therefore, it is extremely useful to search simple molecules allowing understanding the configuration and the function of some complex macromolecules. The title compound, was prepared as part of our ongoing studies of hydrogen-bonding interactions in the crystal structure of protonated amines (Hamdi *et al.*, 2014). Structures containing the 3-chloro-2-methylanilinium cation have been already reported with dihydrogenphosphate (Khemiri *et al.*, 2008) and cyclohexaphosphate (Bel Haj Salah *et al.*, 2014).

The asymmetric unit of the title compound (I), illustrated in Fig. 1, consists of one organic cation, one Cl^-^ anion and one water molecule. All bond distances and angles are within the ranges of accepted values. Moreover, they are close to respect the geometrical features observed in the crystal structure of 5-chloro-2-methylanilinium chloride (Oueslati *et al.*, 2005). Components of the asymmetric unit develop different H-bonds, N—H···Cl, N—H···OW and OW—H···Cl (Fig. 2) that keep them in a state where the aromatic rings of organic buildings are oriented in the direction of the *bc* planes to form centro-symmetric pairs interconnected by hydrogen bonds in the direction of the *b* axis (Fig. 2). The resulting sequences are alternated to form the crystal packing of the title compound (Fig. 3).

### 2. Experimental

An aqueous solution of AlCl_3_ (1 mmol) and 3-chloro-2-methylaniline (2 mmol) in hydrochloric acid was stirred for several minutes at room temperature and slowly evaporated to dryness for two weeks. White single crystals of the title compound were carefully isolated under polarizing microscope for X-ray diffraction analysis.

### 3. Refinement

All H atoms were placed in geometrically idealized positions and constrained to ride on their parent atoms with C—H distances of 0.93 Å for phenyl and 0.96 Å for methyl groups, and N—H = 0.89 Å; *U*_iso_ (H) = 1.2 *U*_iso_ (C,*N*) for phenyl and ammonium H atoms and 1.5 *U*_iso_ (C) for methyl. In water molecule the O—H distances were restrained to 0.85 (1) Å, and the distance H···H to 1.44 (2) Å.

### Crystal data

**Table:**

C_7_H_9_ClN^+^·Cl^−^·H_2_O	*F*(000) = 408
*M**_r_* = 196.07	*D*_x_ = 1.396 Mg m^−^^3^
Orthorhombic, *P*2_1_2_1_2_1_	Ag *K*α radiation, λ = 0.56087 Å
Hall symbol: P 2ac 2ab	Cell parameters from 25 reflections
*a* = 7.434 (4) Å	θ = 9–11°
*b* = 7.475 (3) Å	µ = 0.33 mm^−^^1^
*c* = 16.785 (2) Å	*T* = 293 K
*V* = 932.7 (6) Å^3^	Rectangular, colorless
*Z* = 4	0.50 × 0.25 × 0.15 mm

### Data collection

**Table:**

Enraf–Nonius CAD-4 diffractometer	*R*_int_ = 0.035
Radiation source: fine-focus sealed tube	θ_max_ = 28.0°, θ_min_ = 2.4°
Graphite monochromator	*h* = −3→12
non–profiled ω scans	*k* = −3→12
5019 measured reflections	*l* = −2→28
3947 independent reflections	2 standard reflections every 120 min
1804 reflections with *I* > 2σ(*I*)	intensity decay: 6%

### Refinement

**Table:**

Refinement on *F*^2^	Secondary atom site location: difference Fourier map
Least-squares matrix: full	Hydrogen site location: inferred from neighbouring sites
*R*[*F*^2^ > 2σ(*F*^2^)] = 0.055	H-atom parameters constrained
*wR*(*F*^2^) = 0.163	*w* = 1/[σ^2^(*F*_o_^2^) + (0.0841*P*)^2^] where *P* = (*F*_o_^2^ + 2*F*_c_^2^)/3
*S* = 0.92	(Δ/σ)_max_ < 0.001
3947 reflections	Δρ_max_ = 0.35 e Å^−^^3^
100 parameters	Δρ_min_ = −0.42 e Å^−^^3^
3 restraints	Absolute structure: Flack (1983), unique data only
Primary atom site location: structure-invariant direct methods	Absolute structure parameter: −0.04 (19)

### Special details

**Table:**

Geometry. All e.s.d.'s (except the e.s.d. in the dihedral angle between two l.s. planes) are estimated using the full covariance matrix. The cell e.s.d.'s are taken into account individually in the estimation of e.s.d.'s in distances, angles and torsion angles; correlations between e.s.d.'s in cell parameters are only used when they are defined by crystal symmetry. An approximate (isotropic) treatment of cell e.s.d.'s is used for estimating e.s.d.'s involving l.s. planes.
Refinement. Refinement of *F*^2^ against ALL reflections. The weighted *R*-factor *wR* and goodness of fit *S* are based on *F*^2^, conventional *R*-factors *R* are based on *F*, with *F* set to zero for negative *F*^2^. The threshold expression of *F*^2^ > σ(*F*^2^) is used only for calculating *R*-factors(gt) *etc*. and is not relevant to the choice of reflections for refinement. *R*-factors based on *F*^2^ are statistically about twice as large as those based on *F*, and *R*- factors based on ALL data will be even larger.

### Fractional atomic coordinates and isotropic or equivalent isotropic displacement parameters (Å^2^)

**Table:**

	*x*	*y*	*z*	*U*_iso_*/*U*_eq_	
Cl1	0.21684 (13)	0.68978 (12)	0.14721 (5)	0.0605 (3)	
N1	0.2400 (3)	0.3630 (3)	0.41835 (13)	0.0397 (7)	
C1	0.2542 (4)	0.3587 (4)	0.33084 (16)	0.0375 (8)	
C2	0.2248 (4)	0.5163 (4)	0.28915 (15)	0.0368 (7)	
C3	0.2477 (4)	0.5022 (5)	0.20642 (15)	0.0429 (8)	
C4	0.2959 (5)	0.3453 (4)	0.16946 (19)	0.0520 (10)	
C5	0.3203 (5)	0.1946 (5)	0.2135 (2)	0.0581 (10)	
C6	0.3010 (4)	0.2003 (4)	0.29557 (18)	0.0435 (8)	
C7	0.1793 (5)	0.6906 (4)	0.3283 (2)	0.0551 (10)	
O1W	0.5280 (3)	0.0960 (3)	0.01902 (16)	0.0676 (9)	
Cl2	0.62142 (10)	0.50337 (10)	0.00015 (4)	0.0469 (2)	
H1A	0.20910	0.47250	0.43403	0.0477*	
H1B	0.34559	0.33388	0.43972	0.0477*	
H1C	0.15674	0.28516	0.43419	0.0477*	
H3	0.31954	0.09820	0.32617	0.0522*	
H4	0.35005	0.08749	0.18857	0.0698*	
H5	0.31181	0.34223	0.11452	0.0624*	
H7A	0.16524	0.78143	0.28835	0.0825*	
H7B	0.27430	0.72387	0.36406	0.0825*	
H7C	0.06902	0.67814	0.35757	0.0825*	
H1W1	0.42091	0.05452	0.01130	0.0825*	
H2W1	0.52857	0.20105	−0.00122	0.0825*	

### Atomic displacement parameters (Å^2^)

**Table:**

	*U*^11^	*U*^22^	*U*^33^	*U*^12^	*U*^13^	*U*^23^
Cl1	0.0707 (6)	0.0555 (5)	0.0553 (4)	0.0060 (5)	−0.0007 (4)	0.0165 (4)
N1	0.0420 (14)	0.0319 (11)	0.0452 (11)	0.0055 (11)	0.0059 (11)	0.0037 (9)
C1	0.0382 (16)	0.0326 (12)	0.0417 (12)	0.0018 (12)	0.0008 (12)	−0.0010 (10)
C2	0.0342 (13)	0.0304 (12)	0.0458 (13)	0.0014 (13)	−0.0018 (12)	−0.0004 (11)
C3	0.0385 (16)	0.0449 (14)	0.0452 (13)	0.0025 (16)	0.0000 (12)	0.0064 (14)
C4	0.0593 (19)	0.0548 (19)	0.0419 (14)	0.006 (2)	0.0005 (15)	−0.0039 (14)
C5	0.074 (2)	0.0428 (16)	0.0576 (18)	0.012 (2)	0.0078 (18)	−0.0144 (16)
C6	0.0456 (16)	0.0296 (12)	0.0554 (15)	0.0057 (15)	0.0021 (14)	−0.0023 (13)
C7	0.073 (2)	0.0393 (16)	0.0529 (16)	0.0120 (19)	0.0011 (16)	−0.0014 (16)
O1W	0.0608 (15)	0.0390 (12)	0.103 (2)	−0.0020 (11)	−0.0192 (16)	0.0113 (13)
Cl2	0.0476 (3)	0.0359 (3)	0.0573 (4)	−0.0004 (3)	−0.0068 (4)	0.0006 (4)

### Geometric parameters (Å, º)

**Table:**

Cl1—C3	1.734 (4)	C2—C3	1.403 (4)
O1W—H2W1	0.8600	C3—C4	1.374 (5)
O1W—H1W1	0.8600	C4—C5	1.360 (5)
N1—C1	1.473 (4)	C5—C6	1.386 (5)
N1—H1A	0.8900	C4—H5	0.9300
N1—H1C	0.8900	C5—H4	0.9300
N1—H1B	0.8900	C6—H3	0.9300
C1—C2	1.388 (4)	C7—H7C	0.9600
C1—C6	1.369 (4)	C7—H7A	0.9600
C2—C7	1.498 (4)	C7—H7B	0.9600
			
H1W1—O1W—H2W1	106.00	C3—C4—C5	119.8 (3)
H1A—N1—H1C	109.00	C4—C5—C6	120.1 (3)
H1B—N1—H1C	109.00	C1—C6—C5	118.9 (3)
C1—N1—H1B	109.00	C3—C4—H5	120.00
C1—N1—H1C	109.00	C5—C4—H5	120.00
C1—N1—H1A	109.00	C6—C5—H4	120.00
H1A—N1—H1B	109.00	C4—C5—H4	120.00
C2—C1—C6	123.8 (3)	C1—C6—H3	121.00
N1—C1—C2	118.2 (2)	C5—C6—H3	121.00
N1—C1—C6	117.9 (3)	C2—C7—H7B	109.00
C1—C2—C7	123.6 (2)	C2—C7—H7C	109.00
C3—C2—C7	121.8 (3)	C2—C7—H7A	109.00
C1—C2—C3	114.6 (3)	H7A—C7—H7C	109.00
Cl1—C3—C4	117.8 (2)	H7B—C7—H7C	109.00
C2—C3—C4	122.9 (3)	H7A—C7—H7B	109.00
Cl1—C3—C2	119.4 (3)		
			
N1—C1—C2—C3	177.7 (3)	C1—C2—C3—C4	−0.2 (5)
N1—C1—C2—C7	0.2 (4)	C7—C2—C3—Cl1	−1.8 (4)
C6—C1—C2—C3	−0.3 (4)	C7—C2—C3—C4	177.6 (3)
C6—C1—C2—C7	−178.0 (3)	Cl1—C3—C4—C5	−179.5 (3)
N1—C1—C6—C5	−178.1 (3)	C2—C3—C4—C5	1.2 (5)
C2—C1—C6—C5	−0.2 (5)	C3—C4—C5—C6	−1.6 (5)
C1—C2—C3—Cl1	−179.5 (2)	C4—C5—C6—C1	1.1 (5)

### Hydrogen-bond geometry (Å, º)

**Table:**

*D*—H···*A*	*D*—H	H···*A*	*D*···*A*	*D*—H···*A*
O1*W*—H1*W*1···Cl2^i^	0.8600	2.2800	3.129 (3)	169.00
O1*W*—H2*W*1···Cl2	0.8600	2.3600	3.139 (3)	151.00
N1—H1*A*···Cl2^ii^	0.8900	2.7000	3.178 (3)	115.00
N1—H1*A*···O1*W*^iii^	0.8900	2.3000	2.667 (4)	105.00
N1—H1*B*···Cl2^iv^	0.8900	2.6800	3.187 (3)	117.00
N1—H1*B*···O1*W*^iii^	0.8900	2.2800	2.667 (4)	106.00
N1—H1*C*···Cl2^ii^	0.8900	2.8300	3.178 (3)	105.00
C7—H7*A*···Cl1	0.9600	2.5000	3.053 (4)	117.00

Symmetry codes: (i) *x*−1/2, −*y*+1/2, −*z*; (ii) −*x*+1/2, −*y*+1, *z*+1/2; (iii) −*x*+1, *y*+1/2, −*z*+1/2; (iv) −*x*+1, *y*−1/2, −*z*+1/2.
